# Multiple myeloma presenting with cavernous sinus syndrome: A case report and literature review

**DOI:** 10.1177/2050313X251412717

**Published:** 2026-09-23

**Authors:** Yusuf Hallak, Mohamed Malek Soumakieh, Bisher Sawaf, Geiger Matthew, Ahmad BaTheeb, Ashraf I. Ahmed, Nezam Altorok, Mani Askari

**Affiliations:** 1 Department of Internal Medicine, University of Toledo Medical Center, OH, USA; 2 Boonshoft School of Medicine, Wright State University, Dayton, OH, USA; 3 Department of Internal Medicine, Hamad Medical Cooperation, Doha, Qatar; 4 College of Medicine, QU health, Qatar University, Doha, Qatar

**Keywords:** cavernous sinus syndrome, extramedullary myeloma, intracranial tumors, multiple myeloma, malignancy, case report

## Abstract

A 68-year-old man presented with progressive left cranial nerve III palsy, initially misattributed to a vascular etiology. Early magnetic resonance imaging revealed a mass in the left cavernous sinus with extensive calvarial lesions. Concurrent serum studies, including serum protein electrophoresis and free light-chain assays, identified hypercalcemia, renal dysfunction, anemia, bone lesions features, and a monoclonal Immunoglobulin A lambda spike, leading to a diagnosis of multiple myeloma with central nervous system involvement manifesting as cavernous sinus syndrome (CSS). The patient responded rapidly to corticosteroids and palliative radiotherapy, followed by systemic chemotherapy. This case illustrates the critical role of early neuroimaging and comprehensive serum studies in diagnosing CSS due to multiple myeloma and emphasizes the need for heightened clinical suspicion when evaluating atypical cranial neuropathies.

## Key clinical message

In older adults presenting with progressive cranial neuropathies, early use of MRI and serum protein studies (SPEP, light chains) is essential to uncovering rare but serious causes such as CNS-involved multiple myeloma, enabling faster diagnosis and treatment.

## Introduction

Multiple myeloma (MM) is a malignant plasma cell disorder characterized by clonal proliferation of plasma cells in the bone marrow, leading to a spectrum of clinical manifestations, including hypercalcemia, renal dysfunction, anemia, and bone lesions – collectively remembered by the acronym hypercalcemia, renal dysfunction, anemia, and bone lesions.^1^ While the disease predominantly affects the axial skeleton, cranial involvement, particularly of the cavernous sinus, is exceedingly rare and often poses a diagnostic challenge due to its overlap with other skull base pathologies.^2^

Cavernous sinus syndrome (CSS) presents as a constellation of neurological deficits affecting cranial nerves (CNs) III, IV, V (V1/V2), and VI, often manifesting as ophthalmoplegia, ptosis, facial sensory loss, and diplopia.^3^ Common etiologies include neoplastic, infectious, inflammatory, and vascular causes. Among neoplastic sources, metastatic lesions, lymphomas, and primary meningiomas are more frequently encountered than plasma cell neoplasms.^4^

Involvement of the cavernous sinus by MM may occur through direct extension of plasmacytomas or as part of systemic disease. However, such cases are exceptionally rare and often reported as isolated events in the literature.^5^ Early recognition is critical, as misdiagnosis or delayed treatment may result in irreversible cranial nerve dysfunction and compromised quality of life.

This report describes a rare and diagnostically challenging case of multiple myeloma presenting initially as CSS, highlighting the importance of considering plasma cell dyscrasias in the differential diagnosis of skull base syndromes. A comprehensive review of the literature is also provided to contextualize the clinical presentation and management strategies.

## Case presentation

### Case history and examination

A 68-year-old male with a medical history of hypertension, gastroesophageal reflux disease, and prior shoulder surgeries presented to the emergency department with a 3-week history of persistent headache, accompanied by dizziness, constipation, fatigue, nausea, and vomiting. Notably, he reported a 3-month history of intermittent diplopia and progressive left-sided ptosis that had been worsening over the preceding 2 months. The patient had been evaluated by primary care physician without a definitive diagnosis. He was eventually referred to an ophthalmologist, who noted a subtle evidence of left cranial nerve III (oculomotor) palsy in the context of progressively worsening vertigo and headaches over several weeks. The patient also reported binocular horizontal diplopia that was present constantly, especially while reading or looking into the distance, and worsened in downward and leftward gaze. He described accompanying pain behind the left eye, a sensation of it “popping out of the socket,” and episodic dizziness described as feeling “drunk.”

On examination, there was left-sided ptosis, impaired upward and medial gaze, and mild anisocoria, with the left pupil slightly larger but reactive to light, suggesting a partial, pupil-involving left CN III palsy. No other acute focal deficits were documented. Given the evolving symptoms, localized cranial neuropathy, and vascular risk factors including hypertension, diabetes, hyperlipidemia, and a prior ischemic stroke in 2020, a posterior communicating artery aneurysm was considered a possible compressive etiology. The ophthalmologist recommended urgent neurovascular imaging (Computed Tomography Angiography or Magnetic Resonance Angiography) to rule out this potentially life-threatening cause.

### Investigations and treatment

Initial laboratory evaluation revealed a normocytic anemia with hemoglobin at 9.6 g/dL and thrombocytopenia with platelet count of 117 × 10⁹/L. Severe hypercalcemia was evident with serum calcium at 16.3 mg/dL. Additional abnormalities included elevated total protein (9.0 g/dL), acute kidney injury with serum creatinine of 3.1 mg/dL (baseline 0.76 mg/dL), and elevated blood urea nitrogen at 46 mg/dL. Serum potassium was mildly decreased at 3.4 mmol/L. The Erythrocyte sedmentation rate was elevated at 38 mm/h, while liver function tests and alkaline phosphatase (52 U/L) were within normal limits.

A non-contrast computed tomography (CT) scan of the brain revealed a 3.2 cm expansile soft tissue mass in the left cavernous sinus, without evidence of hemorrhage. Multiple osseous lucent lesions were also noted. Brain magnetic resonance imaging (MRI) further characterized the lesion as contrast-enhancing, involving the left cavernous sinus and accompanied by innumerable calvarial lesions, suggestive of a disseminated skeletal pathology (Figure 1). A chest radiograph showed no acute cardiopulmonary findings.

**Figure 1.**
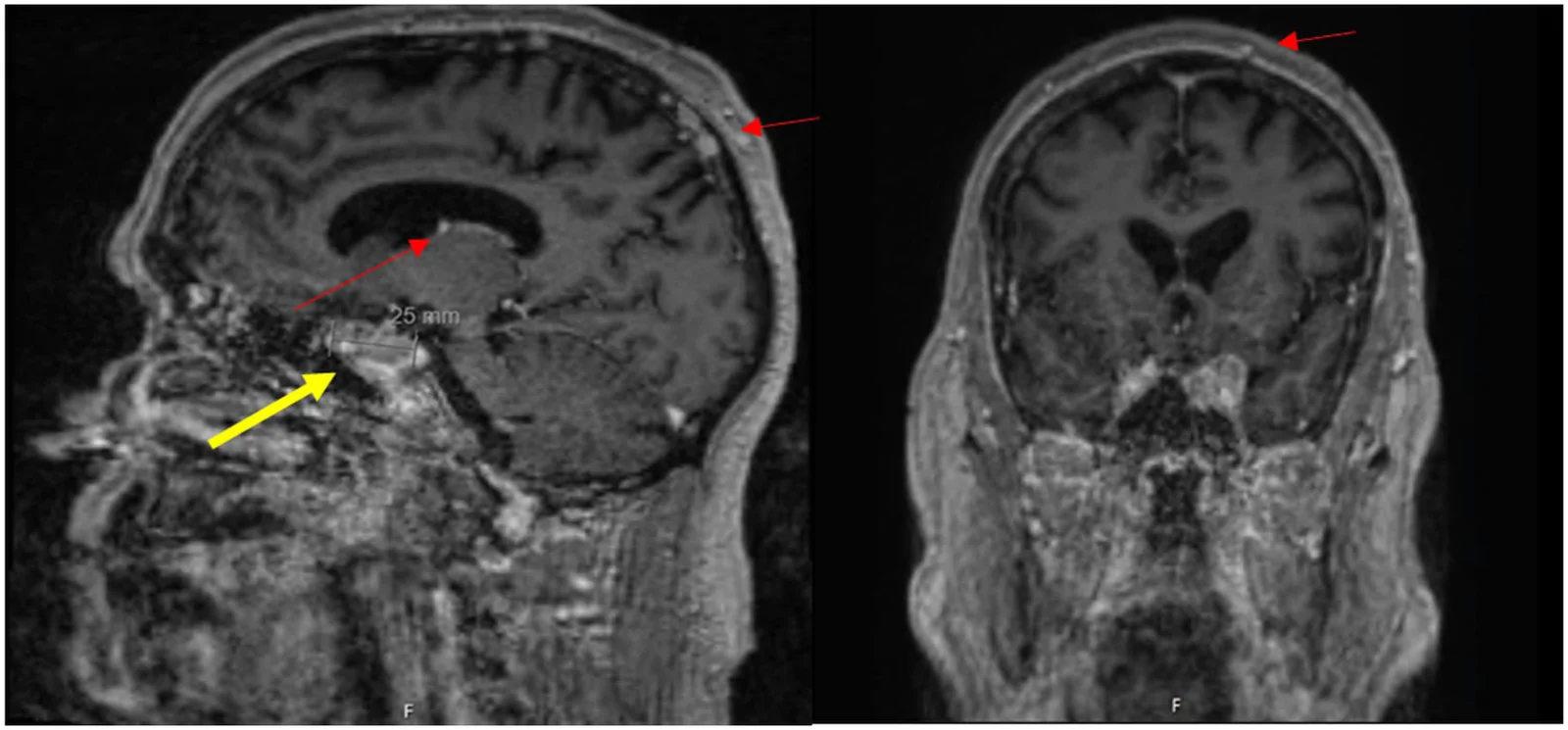
Post-contrast T1-weighted MRI brain showing multiple hypercellular calvarial lesions (red arrows) and a prominent enhancing lesion centered within the left cavernous sinus (yellow arrow). MRI: Magnetic resonance imaging.

Based on the constellation of clinical and radiologic findings, hypercalcemia, lytic bone lesions, acute kidney injury, and anemia, multiple myeloma was strongly suspected. Serum immunoglobulin studies demonstrated markedly elevated Immunoglobulin A (IgA) (3,355 mg/dL), with suppressed Immunoglobulin G (344 mg/dL) and Immunoglobulin M (34 mg/dL), consistent with IgA lambda monoclonal gammopathy. Oncology was consulted and promptly initiated high-dose intravenous dexamethasone therapy. A bone marrow biopsy was ordered to confirm the suspected diagnosis of a plasma cell neoplasm. Supportive measures for hypercalcemia included intravenous fluids, bisphosphonates, and calcitonin. In the setting of markedly elevated serum uric acid (11.7 mg/dL) and concern for tumor lysis syndrome due to a high tumor burden, rasburicase was administered.

Neurosurgical consultation was obtained for a left cavernous sinus mass, but surgical resection was deferred due to the lesion’s critical location and the patient’s systemic disease burden. Palliative radiotherapy was initiated, delivering a total dose of 2000 cGy in 8 planned fractions to the cavernous sinus and other osseous disease sites, including the sternum, left pubic ramus, left femur, and right sacroiliac region, using tailored techniques such as en face electron beams and cone beam CT-guided boosts.

Bone marrow biopsy confirmed the diagnosis of plasma cell myeloma, International Staging system (ISS) Stage III, with elevated β2-microglobulin (11.5 mg/L) and low albumin (2.8 g/dL). The patient was transitioned to systemic therapy with VRD-daratumumab (bortezomib, lenalidomide, and daratumumab), and began Revlimid 25 mg daily on a 2-weeks-on/1-week-off cycle. Concurrent prophylactic and supportive medications included valacyclovir, aspirin, gabapentin, and zoledronic acid. A Positron Emission Tomography scan was ordered to monitor treatment response, and follow-up with oncology and imaging was arranged to guide ongoing care.

### Outcome and follow-up

Following inpatient management, the patient’s hypercalcemia resolved, and significant improvement in headache and left-sided cranial nerve dysfunction was observed, attributed to corticosteroid therapy and radiotherapy. He was discharged with resolution of acute symptoms and was transitioned to outpatient treatment with lenalidomide, bortezomib, dexamethasone, and acyclovir for prophylaxis. The patient’s cranial neuropathy, including headache and facial droop showed marked improvement over the following weeks. Pain management was achieved with prescribed opioid analgesics as needed. Follow-up was arranged for continued oncology care and assessment of therapeutic response.

## Discussion

Central nervous system (CNS) involvement in multiple myeloma is an uncommon but clinically significant complication, occurring in approximately 1% of all MM cases.^6^ At the time of initial diagnosis, CNS involvement is even rarer, with reported rates between 0.1% and 0.5%.^7,8^ Incidence rises to 1%–2% in relapsed or refractory settings, often in association with high-risk cytogenetic abnormalities, extramedullary disease, and prior intensive treatment.^7,8^ Against this epidemiologic backdrop, the present case illustrates the diagnostic challenges posed when MM presents atypically through neurologic symptoms rather than its more familiar skeletal or systemic manifestations.

Timely intervention was essential to prevent irreversible neurologic damage. The therapeutic approach in this case prioritized prompt neurologic symptom relief and systemic disease control. High-dose corticosteroids were first administered for their rapid cytoreductive effect, crucial for mitigating mass effect on cranial nerves. Palliative radiotherapy, delivered at a total dose of 2000 cGy over 8 fractions to the cavernous sinus, provided localized disease control and led to significant improvement in cranial nerve function, reflecting the well-documented radiosensitivity of CNS plasmacytomas. Following clinical stabilization, systemic chemotherapy with a lenalidomide, bortezomib, and dexamethasone regimen was initiated, consistent with the International Myeloma Working Group (IMWG) guidelines.^9,10^ Surgical intervention was deemed inappropriate given the lesion’s deep-seated location and systemic skeletal involvement, affirming that a non-invasive therapeutic strategy was justified and preferable.

Notably, the IMWG and other major bodies currently lack formal guidance on the management of CNS involvement in MM, highlighting a pressing need for consensus recommendations in this rare clinical context. Emerging immunotherapies, including B-cell Maturation Antigen-directed CAR T-cell therapies (idecabtagene vicleucel, ciltacabtagene autoleucel) and bispecific antibodies such as teclistamab and elranatamab, have shown promising activity in relapsed or refractory myeloma, with early reports of efficacy in CNS disease.^11–13^ These agents may overcome CNS sanctuary barriers, though further data are needed. Close monitoring for cytokine release and neurotoxicity remains essential. B-cell Maturation Antigen

Understanding the biological underpinnings of CNS involvement in MM further emphasizes the urgency of early recognition and aggressive treatment. Its pathophysiology is multifactorial, often associated with high-risk cytogenetic abnormalities, such as chromosome 13 deletions, and plasmablastic morphology, both indicators of rapid proliferation and poor prognosis.^14,15^ Extramedullary involvement, particularly soft tissue plasmacytomas and plasma cell leukemia, further correlates with higher tumor burden and increased risk of CNS infiltration.^6^ Malignant plasma cells breach the blood-brain barrier through cytokine-mediated disruption of its integrity, establishing CNS disease in a sanctuary site less accessible to systemic therapies and contributing to treatment resistance and disease progression.^16,17^

This case also reflects broader challenges and insights from the literature on CSS secondary to MM. Across published reports, symptoms such as diplopia, ptosis, ophthalmoplegia, and isolated cranial nerve III and VI palsies predominate, typically developing insidiously over weeks. The overlap with vascular or inflammatory etiologies frequently delayed diagnosis, as highlighted by cases such as those reported by Lam et al. and Silverman et al.,^18,19^ where misattribution to benign causes led to rapid deterioration or death. In many instances, delayed diagnosis reflected a failure to integrate subtle clinical clues with early systemic evaluation, reinforcing the importance of maintaining a broad differential when evaluating isolated cranial neuropathies.

Radiologically, MRI played a central role across the literature, consistently revealing cavernous sinus or adjacent skull base lesions, often accompanied by lytic bone destruction. However, despite characteristic imaging, many cases required histopathologic confirmation via biopsy to achieve a definitive diagnosis. Notably, some cases, such as that described by Lam et al.,^18^ were only diagnosed post-mortem, underscoring the potential for misdiagnosis when clinical vigilance is lacking. In contrast, the present case was diagnosed non-invasively, through the synthesis of imaging features and systemic hematologic profiles, thus avoiding the risks associated with neurosurgical intervention.

Treatment strategies varied widely across reported cases, reflecting institutional preferences and clinical state at diagnosis. Radiotherapy, administered either conventionally or via stereotactic techniques such as gamma knife radiosurgery, consistently emerged as a cornerstone for local disease control. For instance, Peker et al.^20^ demonstrated effective tumor regression with gamma knife radiosurgery combined with systemic therapy. However, systemic chemotherapy yielded mixed outcomes, with some patients, like those described by Sekiguchi et al.,^21^ achieving partial remission, while others, such as Kane’s patient (2024), demonstrated progressive neurological deterioration despite aggressive systemic therapy. In Dhanani’s 2024 series, palliative radiotherapy achieved symptom resolution in most patients, although the prognosis remained guarded due to advanced disease burden.^22^

Outcomes across cases were heterogeneous but generally poor, with mortality frequently occurring within 3–9 months of diagnosis. Particularly dismal prognoses were observed in patients with multifocal extramedullary disease or in those where diagnosis was delayed. Conversely, a minority of patients achieved prolonged remission, suggesting that early recognition and prompt multimodal therapy, integrating both local and systemic treatments, can favorably influence disease trajectory. Review of the available case reveals several consistent themes. First, most patients presented with cranial nerve III and VI palsies, often accompanied by diplopia and ptosis. Second, MRI was almost universally effective in identifying soft tissue lesions involving the cavernous sinus or skull base, highlighting its central role in early diagnosis. Third, combined corticosteroid therapy and localized radiotherapy provided symptomatic relief in over 70% of cases, underscoring the radiosensitivity of CNS-involved myeloma. Despite these interventions, overall prognosis remains poor, with a median survival of less than 6 months from diagnosis in most reported cases (Table 1). Collectively, the literature and this case affirm that while CSS secondary to MM is rare, it constitutes a clinical entity with potentially severe neurologic consequences and poor prognosis if misdiagnosed or managed suboptimally. This case reinforces the necessity of integrating imaging with systemic laboratory evaluation at an early stage. High-resolution contrast-enhanced MRI of the brain and skull base, coupled with basic blood work – including complete blood count, renal and liver panels, calcium levels, and inflammatory markers – should be obtained promptly in patients presenting with isolated cranial nerve palsies. Crucially, serum protein electrophoresis (SPEP) with immunofixation and serum free light-chain assays must be performed early to screen for plasma cell dyscrasias, even in the absence of overt systemic symptoms.

**Table 1.** Summary of key characteristics from the published case reports/series.

Author/Year	Age/Sex	Presenting symptoms	MRI/imaging findings	Treatment given	Outcome
Selçuk Peker et al.^20^	70/F	Headache, diplopia, left earache, hypoesthesia (CN V), CN VI palsy	Plasmacytoma in left cavernous sinus, IgG lambda MM confirmed, no bone lesions	Gamma knife radiosurgery (14 Gy), prednisolone + melphalan	Complete tumor disappearance on MRI; patient died of sepsis 26 months later
Katsunori Kyoda et al.^23^	53/M	Diplopia, infraorbital/zygomatic pain, CN V and VI palsy	Plasmacytoma in sphenoid sinus, IgG positive, nonsecretory MM with CNS involvement	Mild response to chemo (Adriamycin, vincristine, dexamethasone)	Died 9 months post RT from leukemic progression
Yasunobu Sekiguchi et al.^21^	56/M	Epistaxis, hoarseness, swelling of finger	Extramedullary plasmablastic plasmacytoma, CD45+, MPC-1−, CD49e−, IgGk type	Thalidomide + Dexamethasone (7 months partial remission)	Recurrence, treated with Bortezomib; patient died of multiple organ failure
Joseph Kane, OD, FAAO^24^	74/M	Ptosis, diplopia, eye ache, CN III & VI palsy	1.3 × 2.2 cm right cavernous sinus lesion, IgG kappa MM, lesion likely myelomatous	No significant improvement; progressive CN palsies	Death 3 months after symptom onset
Lam S, Margo CE, Beck R, et al.^18^	76/M	Diplopia, mild ptosis, CN III and VI palsy	Plasmacytoma in cavernous sinus (found at autopsy), diagnosis post-mortem	None (misdiagnosis)	Patient died 3 months after presentation
Rebecca F. Silverman et al.^19^	60/M	Diplopia, ptosis, ophthalmoplegia, left eye mydriasis	Cavernous sinus plasmacytoma, IgG lambda light-chain MM, FGFR3-IGH [*t*(4;14)]	Planned bortezomib (delayed), patient deteriorated rapidly	Expired 5 months after diagnosis due to systemic complications
Eilis Fitzgerald et al.^25^	71/F	Diplopia, CN VI palsy	Diffuse leptomeningeal enhancement, CNS myelomatosis from IgG kappa MM; nonsecretory MM, plasmacytoma in sphenoid sinus extending to sella, 70% marrow plasma cells	Chemotherapy (adriamycin, vincristin, dexamethasone); none (rapid decline)	Death 5 weeks after onset of visual symptoms; patient died 9 months post RT; autopsy not performed
Chiranjiwi Prasad Shah et al.^26^	68/M	Blurry vision, periorbital pain, ptosis, right CN III, IV, VI palsy	5.8 × 3.6 × 4.2 cm clival mass extending to sphenoid and ethmoid sinuses; biopsy showed plasmacytosis (58%)	Cyclophosphamide, bortezomib, dexamethasone (VCD)	Significant improvement in extraocular motility; visual acuity improved
Alessandro Gozzetti et al.^27^	62/F, 80/F, 82/M	Diplopia, ptosis, orbital pain, CN III palsy	Cavernous sinus involvement via clivus/dural disease in MM relapse or first presentation	Bortezomib ± dexamethasone (partial/complete neurologic response)	One patient remained well, one relapsed systemically, one died post RT
Anne Soejbjerg et al.^28^	62/F	Blurred vision, temporal pain (initially diagnosed as temporal arteritis)	Sellar mass encasing right carotid artery, histology showed kappa monoclonal plasmacytoma	Post-op radiation (46 Gy over 5 weeks)	Remained in remission 3.5 years post treatment; developed central diabetes insipidus
Aaron R. Kaufman et al.^29^	44/M	Acute diplopia, left CN VI palsy, positional headache	Clival mass (4.3 × 1.8 × 2.3 cm), hypermetabolic, nonsecretory MM relapse confirmed via sternal biopsy	Daratumumab + bortezomib; marked response in clival lesion	Complete recovery of eye movement in 7 months; mixed systemic response
Chunyan Li et al.^30^	47/F	Right ptosis and diplopia progressing to bilateral ptosis and numbness	Bilateral cavernous sinus masses, leptomeningeal nodules, bone and organ metastases; confirmed MM (IgG κ type)	Bortezomib, dexamethasone, and thalidomide	Initial improvement, but patient died from severe lung infection 1 month later
Federico L. Ampil et al.^31^	63/M	Retro-orbital headache, diplopia, CN VI palsy	EMP of sphenoid sinus extending into cavernous sinus; biopsy confirmed plasmacytoma	Post-RT chemotherapy with melphalan and prednisone	Minimal response; recurrence required surgery; persistent disease despite therapy
I. Vande Broek et al.^32^	80/M	Diplopia, exophthalmia, ptosis, orbital pain	Mass in right cavernous sinus; relapsed MM with monoclonal peak, no bone marrow plasmacytosis	None (no response)	Death within 1 week of admission
Martin Galea et al.^33^	58/F	Vertical diplopia, variable left upper lid ptosis	Frontal bone lesion, confirmed light-chain MM with widespread bone lesions	Cyclophosphamide, Thalidomide, and Dexamethasone	Not specified
AI-Noor N. Dhanani et al.^34^	62/M	Diplopia, headaches, left CN VI palsy	Sellar mass (2.4 cm) extending into sphenoid sinus; biopsy confirmed plasmacytoma; bone marrow 20%–100% plasma cells	Chemotherapy for MM post surgery	Alive at 6 months; L2 fracture and hypercalcemia developed
D. Wachter et al.^35^	43/F	Progressive then acute unilateral visual loss (left eye)	3 cm orbital apex mass encasing optic nerve; biopsy confirmed lambda + plasmacytoma; vertebral involvement	RT and intrathecal methotrexate, later chemotherapy	Vision improved; systemic MM treatment initiated
Nishanth Thalambedu et al.^36^	55/M and 69/M	Double vision, right CN VI palsy (Case 1); Diplopia, left CN VI palsy (Case 2)	Case 1: Right jugular foramen mass with clival erosion; Case 2: Mass in left petrous apex and cavernous sinus	Case 1: KDT-PACE chemotherapy; Case 2: Blenrep (belantamab mafodotin)	Both patients had resolution of diplopia and stable/improving systemic disease
Ujalashah Dhanani et al.^22^	7 patients (42–71 years, M/F)	Diplopia, ptosis, ophthalmoplegia, CN palsies (III, IV, VI), orbital pain, proptosis	Lesions in superior orbital fissure, cavernous sinus, sphenoid sinus; extramedullary myeloma	Radiation effective in most cases; some received additional chemotherapy	Diplopia resolved in majority; two patients died from MM complications

CD, cluster of differentiation; CN, cranial nerve; CNS, central nervous system; EMP, extramedullary plasmacytoma; FAAO, fellow of the American Academy of Optometry; FGFR3-IGH, fibroblast growth factor receptor 3–immunoglobulin heavy chain gene rearrangement [t(4;14)]; IgG, immunoglobulin G; KDT-PACE, carfilzomib, dexamethasone, thalidomide, cisplatin, doxorubicin (Adriamycin), cyclophosphamide, and etoposide; MM, multiple myeloma; MPC, mature plasma cell antigen; MRI, magnetic resonance imaging; OD, doctor of optometry; RT, radiotherapy; VCD, bortezomib (Velcade), cyclophosphamide, dexamethasone.

## Conclusion

CNS involvement in multiple myeloma is a rare but highly aggressive complication associated with poor outcomes if not diagnosed and treated expeditiously. This case highlights the diagnostic challenges posed by atypical neurologic presentations, emphasizing the importance of maintaining a broad differential diagnosis for isolated cranial neuropathies. Early integration of high-resolution neuroimaging and systemic laboratory studies, including screening for monoclonal gammopathies, is critical in guiding timely diagnosis. Management strategies combining corticosteroids, radiotherapy, and systemic chemotherapy remain the foundation of care, while emerging immunotherapeutic options such as CAR T-cell therapies and bispecific antibodies offer promising avenues for future treatment, even in CNS-involved disease. Broader recognition of CNS-MM and the development of consensus guidelines are essential to improving prognosis for this challenging clinical entity. Early suspicion, rapid diagnostic workup, and multidisciplinary intervention remain the key pillars to achieving better outcomes.

## Data Availability

The datasets generated during and/or analyzed during the current study are available from the corresponding author upon reasonable request.

## References

[1] Rajkumar SV Dimopoulos MA Palumbo A , et al. International Myeloma Working Group updated criteria for the diagnosis of multiple myeloma. Lancet Oncol 2014; 15(12): e538–e548. 10.1016/S1470-2045(14)70442-5 25439696

[2] Kyle RA Rajkumar SV. Multiple myeloma. N Engl J Med 2004; 351(18): 1860–1873. 10.1056/NEJMra041875 15509819

[3] Nambiar R Nair SG. Cavernous sinus syndrome. Proc (Bayl Univ Med Cent) 2017; 30(4): 455–456. 10.1080/08998280.2017.11930227 PMC5595394 28966464

[4] Toro J Burbano LE Reyes S , et al. Cavernous sinus syndrome: need for early diagnosis. BMJ Case Rep 2015; 2015: bcr2014206999. 10.1136/bcr-2014-206999 PMC4386434 25819816

[5] Lasocki A Gangatharan S Gaillard F , et al. Intracranial involvement by multiple myeloma. Clin Radiol 2015; 70(8): 890–897. 10.1016/j.crad.2015.03.014 25956664

[6] Katodritou E Dalampira D Delimpasi S , et al. Central nervous system multiple myeloma: a real-world multi-institutional study of the Greek Myeloma Study Group. Am J Hematol 2024; 99(10): 1897–1905. 10.1002/ajh.27425 38953456

[7] Yamashita T Takamatsu H Kawamura K , et al. A nationwide survey on central nervous system multiple myeloma in Japan: analysis of prognostic and treatment factors that impact survival. Br J Haematol 2021; 195(2): 217–229. 10.1111/bjh.17717 34318926

[8] Jurczyszyn A Grzasko N Gozzetti A , et al. Central nervous system involvement by multiple myeloma: a multi-institutional retrospective study of 172 patients in daily clinical practice. Am J Hematol 2016; 91(6): 575–580. 10.1002/ajh.24351 PMC5502095 26955792

[9] Richardson PG Durie BG Rosiñol L , et al. Clinical perspectives on the optimal use of lenalidomide plus bortezomib and dexamethasone for the treatment of newly diagnosed multiple myeloma. Haematologica 2023; 108(11): 2894–2912. 10.3324/haematol.2022.282624 PMC10620581 37608773

[10] Joseph NS Kaufman JL Dhodapkar MV , et al. Long-term follow-up results of lenalidomide, bortezomib, and dexamethasone induction therapy and risk-adapted maintenance approach in newly diagnosed multiple myeloma. J Clin Oncol 2020; 38(17): 1928–1937. 10.1200/JCO.19.02515 PMC7587409 32298201

[11] Holstein SA Grant SJ Wildes TM. Chimeric antigen receptor t-cell and bispecific antibody therapy in multiple myeloma: moving into the future. J Clin Oncol 2023; 41(27): 4416–4429. 10.1200/JCO.23.00512 PMC10522112 37471687

[bibr12-2050313X251412717] Mikkilineni L Sidana S. Integrating immune therapies for the treatment of multiple myeloma. J Natl Compr Canc Netw 2023; 21(12): 1303–1311. 10.6004/jnccn.2023.7100 38081142

[13] Abramson HN. Immunotherapy of multiple myeloma: current status as prologue to the future. Int J Mol Sci 2023; 24(21): 15674. 10.3390/ijms242115674 PMC10649824 37958658

[14] Fassas AB Muwalla F Berryman T , et al. Myeloma of the central nervous system: association with high-risk chromosomal abnormalities, plasmablastic morphology and extramedullary manifestations. Br J Haematol 2002; 117(1): 103–108. 10.1046/j.1365-2141.2002.03401.x 11918539

[15] Fassas AB Ward S Muwalla F , et al. Myeloma of the central nervous system: strong association with unfavorable chromosomal abnormalities and other high-risk disease features. Leuk Lymphoma 2004; 45(2): 291–300. 10.1080/10428190310001597964 15101714

[16] Akhtar S Ali TA Faiyaz A , et al. Cytokine-mediated dysregulation of signaling pathways in the pathogenesis of multiple myeloma. Int J Mol Sci 2020; 21(14): 5002. 10.3390/ijms21145002 PMC7403981 32679860

[17] Lee D Kalff A Low M , et al. Central nervous system multiple myeloma–potential roles for intrathecal therapy and measurement of cerebrospinal fluid light chains. Br J Haematol 2013; 162(3): 371–375. 10.1111/bjh.12404 23718539

[18] Lam S Margo CE Beck R , et al. Cavernous sinus syndrome as the initial manifestation of multiple myeloma. J Clin Neuroophthalmol 1987; 7(3): 135–138. 2958504

[19] Silverman RF Hanson L Salahi N , et al. Case report: cavernous sinus syndrome as the initial presentation of multiple myeloma. Front Ophthalmol (Lausanne) 2022; 2: 849343. 10.3389/fopht.2022.849343 PMC11182293 38983538

[20] Peker S Abacioğlu U Bayrakli F , et al. Gamma knife radiosurgery for cavernous sinus plasmacytoma in a patient with breast cancer history. Surg Neurol 2005; 63(2): 174–176; discussion 176–177. 10.1016/j.surneu.2003.12.013 15680666

[21] Sekiguchi Y Asahina T Shimada A , et al. A case of extramedullary plasmablastic plasmacytoma successfully treated using a combination of thalidomide and dexamethasone and a review of the medical literature. J Clin Exp Hematop 2013; 53(1): 21–28. 10.3960/jslrt.53.21 23801130

[22] Dhanani U Mortensen PW Raviskanthan S , et al. Ophthalmoplegia associated with extramedullary multiple myeloma: case series from a tertiary cancer center. J Neuroophthalmol 2024; 44(4): 533–539. 10.1097/WNO.0000000000002076 38175728

[23] Kyoda K Nakamura S Takeshima M , et al. Cavernous sinus syndrome associated with nonsecretory myeloma. Intern Med 1999; 38(12): 988–990. 10.2169/internalmedicine.38.988 10628940

[24] Kane J. Cavernous sinus involvement in multiple myeloma. J Med Optometry, 2024; 2: 3.

[25] Fitzgerald E Kiely P Leary HO. Intracranial involvement in multiple myeloma presenting as a cranial nerve palsy. J Hematol 2019; 8(1): 29–33. 10.14740/jh468 PMC7153671 32300438

[26] Shah CP Chamlagain R Shah S , et al. Multiple myeloma with plasmacytoma of the clivus bone presenting with multiple cranial nerve III, IV, and VI palsy: a diagnostic dilemma. Clin Case Rep 2023; 11(2): e6958. 10.1002/ccr3.6958 PMC9923479 36794041

[27] Gozzetti A Cerase A Tarantino A , et al. Multiple myeloma involving the cavernous sinus: a report of 3 cases and response to bortezomib. Clin Lymphoma Myeloma 2007; 7(5): 376–378. 10.3816/CLM.2007.n.017 17562250

[28] Soejbjerg A Dyve S Baerentzen S , et al. The solitary sellar plasmacytoma: a diagnostic challenge. Endocrinol Diabetes Metab Case Rep 2016; 2016: 160031. 10.1530/EDM-16-0031 PMC4898066 27284454

[29] Kaufman AR Quillen K Distefano AG , et al. Nonsecretory recurrence of multiple myeloma presenting as sixth nerve palsy secondary to clival plasmacytoma. J Neuroophthalmol 2021; 41(1): e77–e78. 10.1097/WNO.0000000000000939 32235225

[30] Li C Shao F Yang Y , et al. Diffuse infiltration of multiple myeloma with initial manifestation of cavernous sinus syndrome unveiled by 18F-FDG PET/CT. Clin Nucl Med 2019; 44(9): 746–747. 10.1097/RLU.0000000000002722 31306198

[31] Ampil FL Borski TG Nathan CO , et al. Cavernous sinus involvement by extramedullary plasmacytoma of the sphenoid sinus. An argument for the use of adjuvant chemotherapy. Leuk Lymphoma 2002; 43(10): 2037–2040. 10.1080/1042819021000015998 12481905

[32] Vande Broek I Stadnik T Meurs A , et al. Extramedullary plasmacytoma of the cavernous sinus. Leuk Lymphoma 2002; 43(8): 1691–1693. 10.1080/1042819021000003054 12400615

[33] Galea M McMillan N Weir C. Diplopia and variable ptosis as the sole initial findings in a case of orbital plasmacytoma and multiple myeloma. Semin Ophthalmol 2015; 30(3): 235–237. 10.3109/13506129.2013.839807 24164055

[34] Dhanani AN Bilbao JM Kovacs K. Multiple myeloma presenting as a sellar plasmacytoma and mimicking a pituitary tumor: report of a case and a review of the literature. Endocr Pathol 1990; 1(4): 245–248. 10.1007/BF02915418 32357616

[35] Wachter D Böker DK Huegens-Penzel M , et al. First manifestation of lambda positive plasmacytoma in the orbital apex with acute unilateral loss of vision. Minim Invasive Neurosurg 2010; 53(2): 74–76. 10.1055/s-0030-1249051 20533138

[36] Thalambedu N Damalcheruvu P Ogunsesan Y , et al. Sixth nerve palsy from multiple myeloma in central nervous system: case series and review of current literature. Cureus 2023; 15(6): e40998. 10.7759/cureus.40998 PMC10371386 37503472

